# Case report: A female case of isolated IgG4-related sclerosing cholangitis mimicking cholangiocarcinoma

**DOI:** 10.1097/MD.0000000000006542

**Published:** 2017-04-21

**Authors:** Jianchun Xiao, Guanqiao Li, Gang Yang, Congwei Jia, Binglu Li

**Affiliations:** a Department of General Surgery; b Department of Pathology, Peking Union Medical College Hospital, Chinese Academy of Medical Sciences, Beijing, China.

**Keywords:** autoimmune biliary disease, bile duct surgery, cholangiocarcinoma

## Abstract

**Rationale:**

IgG4-related disease is a newly recognized fibroinflammatory disorder, characterized by tumefactive lesions, storiform fibrosis and IgG4-positive plasma cells infiltration. IgG4-related sclerosing cholangitis (IgG4-SC) is the most common extrapancreatic manifestation of IgG4-related disease, but it is frequently associated with autoimmune pancreatitis(AIP). Only few case was reported to be diagnosed with IgG4-SC in the absence of AIP, with a striking male preponderance. Here we report a female case of isolated IgG4 related sclerosing cholangitis mimicking cholangiocarcinoma.

**Patient concerns:**

A 58-year-old woman complaint of one-month history of jaundice and right upper quadrant discomfort, and the biliary reconstruction showed full-length wall thickening and segmental stenosis.

**Diagnoses:**

Cholangiocarcinoma was then diagnosed.

**Interventions:**

Choledochoplasty was performed, followed by Roux-en-Y anastomosis.

**Outcomes:**

However, pathological examination revealed IgG4-related sclerosing cholangitis (IgG4-SC) and the retrospective measurement of serum IgG4 was 346 mg/dL post-operatively. The patient was followed for another nine monthswithout recurrence.

**Lessons:**

The differential diagnosis between cholangiocarcinoma and IgG4-SC is challenging due to significant overlap of clinical manifestations, lab tests and imaging characteristics. However, as an afterthought of this case, typical cholangiocarcinoma rarely presents full-length wall thickening. What the case taught us was pre-operative IgG4 measurement for patients with long bile duct involvement was highly recommended in order to rule out IgG4-SC.

## 1 Introduction

IgG4-related disease is a newly recognized fibroinflammatory disorder, which is characterized by tumefactive lesions, storiform fibrosis, IgG4-positive plasma cells infiltration as well as frequent but not always elevated serum IgG4 level.^[1]^ IgG4-related sclerosing cholangitis (IgG4-SC) is the most common extrapancreatic manifestation of IgG4-related disease, and it has become the third distinct disease entity of sclerosing cholangitis.^[2]^ The clinical and cholangiographic abnormalities observed in IgG4-SC may resemble those observed in cholangiocarcinoma. Notably, IgG4-SC frequently keeps accompany with concurrent autoimmune pancreatitis (AIP). Only few cases were reported to be diagnosed with IgG4-SC in the absence of AIP, with a striking male preponderance.^[3]^

## 2 Case history

Written informed consent was obtained, and institutional Ethics Committee of the Peking Union Medical College Hospital approved this case report.

**Figure 1:**
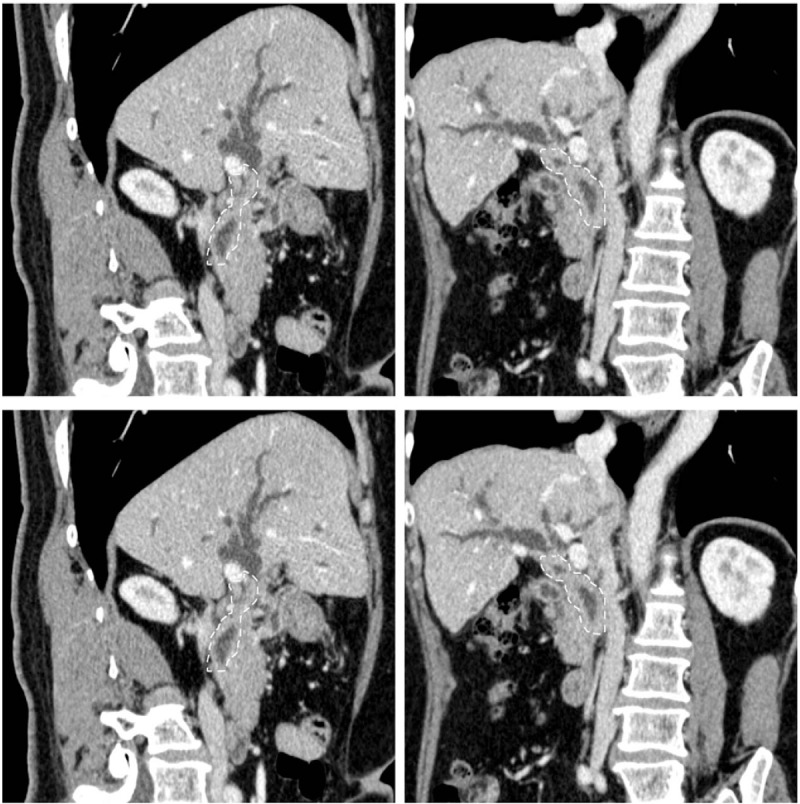
Contrast-enhanced computed tomography with biliary reconstruction showed wall thickening of the entire bile duct and segmental stenosis between 2 dilational parts (dashed line).

**Figure 2:**
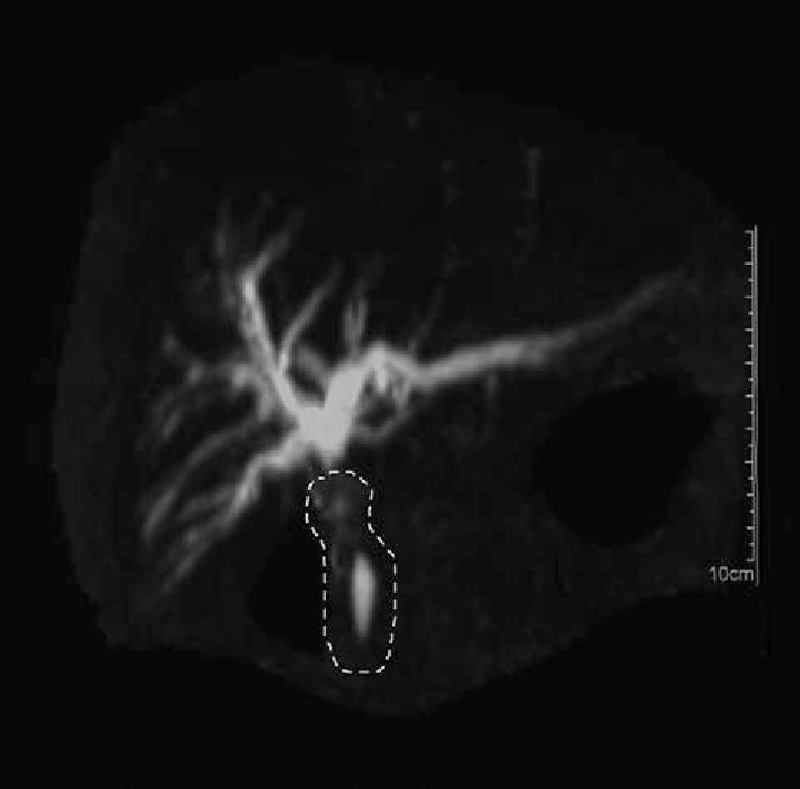
Magnetic resonance cholangiopancreatography showed wall thickening of the entire bile duct and segmental stenosis between 2 dilational parts (dashed line).

**Figure 3:**
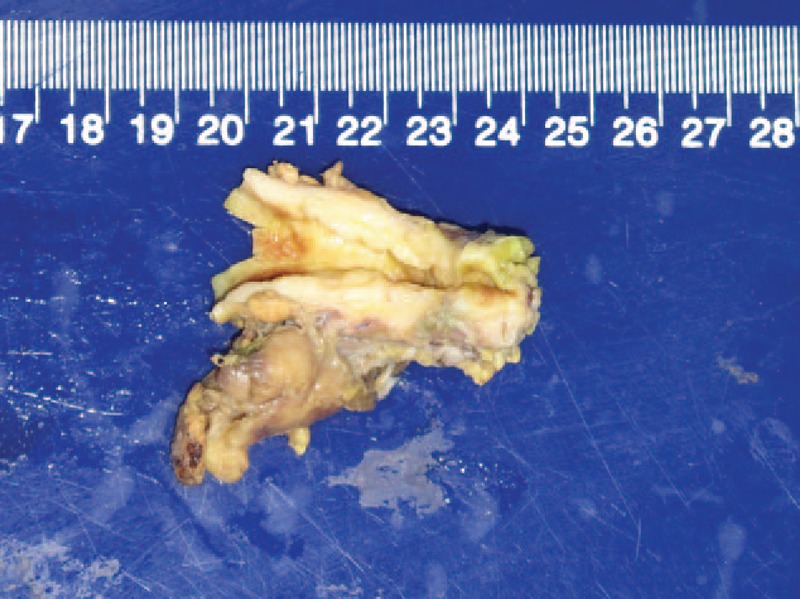
Laparotomy confirmed a coarse texture of the common hepatic duct and a 6-cm-long wall thickening of the proximal bile duct, originating from the start of common hepatic duct to the intrapancreatic bile duct.

**Figure 4:**
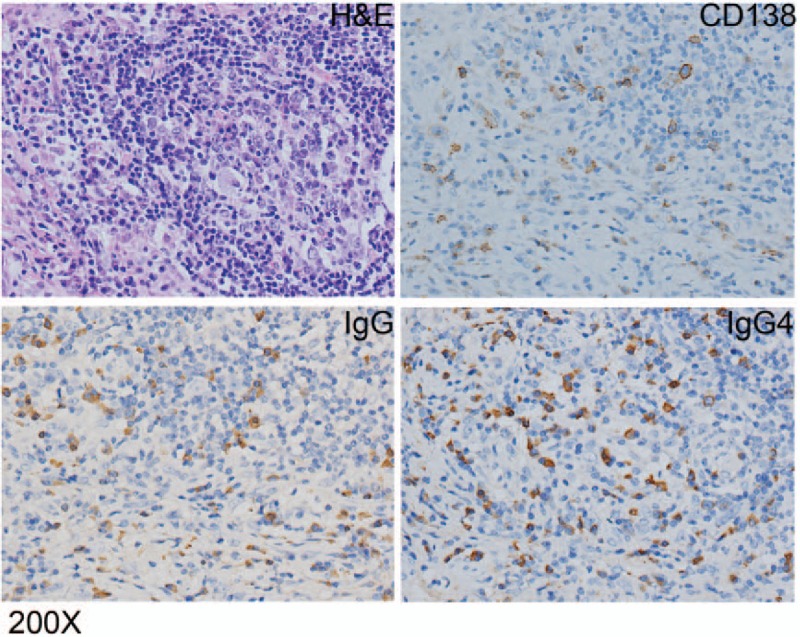
Immunohistochemical staining was positive for CD138, IgG, and IgG4 (IgG4/IgG plasma cell >40%).

A 58-year-old female was admitted to hospital, presented with a 1-month history of jaundice, right upper quadrant discomfort, pruritus, pale stools, and dark urine. Laboratory data revealed: total bilirubin 4.6 mg/dL, direct bilirubin 3.3 mg/dL, alanine aminotransferase 314 U/L, alkaline phosphatase 387 U/L, gamma-glutamyl transpeptidase 1190 U/L, and carbohydrate antigen 19-9 71.7 U/mL. Contrast-enhanced computed tomography with biliary reconstruction showed wall thickening of the entire bile duct and segmental stenosis between 2 dilational parts (Fig. 1, dashed line), confirmed by magnetic resonance cholangiopancreatography (Fig. 2, dashed line). Based on the symptoms and lab data, she was then diagnosed with cholangiocarcinoma. Laparotomy further confirmed a coarse texture of the common hepatic duct and a 6-cm-long wall thickening of the proximal bile duct, originating from the start of common hepatic duct to the intrapancreatic bile duct (Fig. 3). The affected bile duct was excised, followed by the choledochoplasty and Roux-en-Y anastomosis. She recovered well after the surgery, with ameliorated jaundice and normalized liver enzyme levels. However, pathological examination revealed no malignant cells. Instead, plasma cells, lymphocytes infiltrates, and hyperplasia of fibrous tissue were observed in the specimen; immunohistochemical staining was positive for CD138, IgG, and IgG4 (IgG4/IgG plasma cell >40%) (Fig. 4). Hence, IgG4-SC was considered. The retrospective measurement of serum IgG4 was 346 mg/dL postoperatively. The patient was followed for another nine months and no recurrence was reported (Table 1).

**Table 1:**
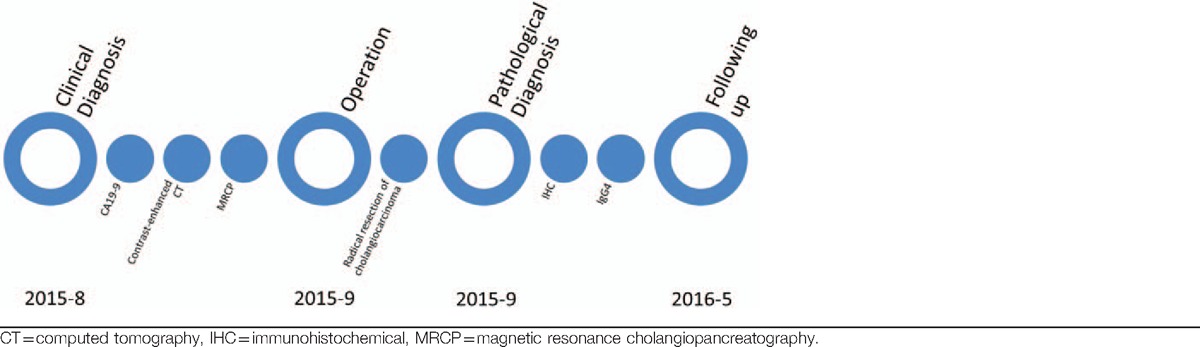
Time line of the case.

## 3 Discussion

The differential diagnosis between cholangiocarcinoma and IgG4-SC is challenging due to significant overlap of clinical manifestations, lab tests, and imaging characteristics. Lacking definitive diagnostic criteria for IgG4-SC, histopathological findings are critical for diagnostic establishment, including dense infiltrates of IgG4+ plasma cells and severe interstitial fibrosis. Furthermore, the presence of increased serum IgG4 concentrations (>135 mg/dL) and involvement of extra-biliary organs also strongly suggest IgG4-SC. Due to the clinical rarity and poor recognition of this entity, it is not surprising that preoperative measurement of serum IgG4 could be missed, especially in those without explicit clinical manifestations in extra-biliary organs.

Without therapeutic guidelines, glucocorticoids remain the first-line agents for symptoms improvement and organ functions restoration.^[2]^ Alternatively, surgical resection was indicated in patients with suspected malignancy, proved to be effective for symptom relief. Notably, relapse occurs in 53% of patients after steroid withdrawal and 44% after surgical intervention.^[4]^

Given the age, gender, clinical, and imaging characteristics of this patient, hilar cholangiocarcinoma was the primary suspect. Endoscopic retrograde cholangiopancreatography might fail to examine the whole bile duct due to the full-length stenosis, so diagnostic laparotomy was performed. However, as an afterthought, typical cholangiocarcinoma rarely presents full-length wall thickening and stenosis of bile duct. Thus we propose preoperative IgG4 measurement for patients presented with long bile duct involvement, and IgG4-SC should be considered if elevated IgG4 exists.
